# Directed Differentiation of Human Embryonic Stem Cells into Corticofugal Neurons Uncovers Heterogeneous *Fezf2*-Expressing Subpopulations

**DOI:** 10.1371/journal.pone.0067292

**Published:** 2013-06-24

**Authors:** Muriel Kmet, Chao Guo, Carina Edmondson, Bin Chen

**Affiliations:** Department of Molecular, Cell and Developmental Biology, University of California Santa Cruz, Santa Cruz, California, United States of America; University of Kansas Medical Center, United States of America

## Abstract

Understanding how neuronal diversity is achieved within the cerebral cortex remains a major challenge in neuroscience. The advent of human embryonic stem cells (hESCs) as a model system provides a unique opportunity to study human corticogenesis *in vitro* and to identify the mechanisms that promote neuronal differentiation to achieve neuronal diversity in human brain. The transcription factor *Fezf2* is necessary and sufficient for the specification of subcerebral projection neurons in mouse. However, its function during human corticogenesis is poorly understood. This study reports the differentiation of a h*Fezf2*-YFP hESC reporter line into corticofugal projection neurons capable of extending axons toward the spinal cord upon transplantation into neonatal mouse brains. Additionally, we show that triple inhibition of the TGFß/BMP/Wnt-Shh pathway promotes the generation of h*Fezf2*-expressing cells *in vitro*. Finally, this study unveils the isolation of two novel and distinct populations of h*Fezf2*-YFP expressing cells reminiscent of the distinct *Fezf2*-expressing neuronal subtypes in the developing mouse brain. Overall our data suggest that the directed differentiation of hESCs into corticofugal neurons provides a useful model to identify the molecular mechanisms regulating human corticofugal differentiation and survival.

## Introduction

Hundreds of different neuronal subtypes are generated in the central nervous system during development. Among these, cortical projection neurons are essential for high order cognitive and sensory functions in the human brain. Within the six-layered cerebral cortex, subcerebral projection neurons are located in layer 5 and extend their axons to the midbrain (corticotectal projection neurons), brain stem (corticobulbar neurons) or spinal cord (corticospinal motor neurons, or CSMNs). CSMNs are clinically important, as their degeneration has been implicated in upper motor neuron diseases such as Amyotrophic Lateral Sclerosis (ALS) [1], [2]. Neurons from layer 6 project their axons to the thalamus (corticothalamic neurons), and together with CSMNs are referred to as corticofugal projection neurons. Due to their high relevance in human diseases, the molecular mechanisms that underlie the specification and differentiation of subcerebral projection neurons have been the subject of intense investigation.

Much progress has been made toward understanding the molecular mechanisms regulating the development of subcerebral projection neurons in mouse. Several transcription factors, including *Fezf2*, *Bcl11b*, *Tbr1*, *Sox5*, and *Bhlhb5* are part of an interconnected gene network that regulates cortical neuron fate specification and differentiation [3]–[9]. Among them, the zinc-finger transcription factor *Fezf2* (Forebrain Embryonic Zinc Finger 2) is both necessary and sufficient for the specification, differentiation and axon targeting of CSMNs in mouse [10]–[12]. During mouse brain development, *Fezf2* is expressed in progenitor cells as early as embryonic (E) day E8.5, and continues to be expressed at high level in subcerebral projection neurons. *Fezf2* null mice show a lack of subcerebral projection neurons and projections to the spinal cord [10], [11]. Strikingly, the mutant neurons adopt the identity of other cortical projection neuron subtypes [4], [10], [11], [13]. Furthermore, misexpression of *Fezf2* in other neuron subtypes directs their axons to project toward the spinal cord [4], [14], [15]. Ultimately, these studies demonstrate that in mouse, the identity and differentiation of subcerebral neurons is achieved through repression of alternate neuronal subtype identities. Despite these advances, the molecular mechanisms regulating the development of human subcerebral neurons have not been directly investigated due to the lack of an appropriate model system in which to study human cortical neuron differentiation. However, the high conservation of FEZF2 protein between mouse and human, and the similar expression patterns of *Fezf2* during human fetal brain development [16], [17] suggest that *Fezf2* is a specific marker for human subcerebral neurons.

The cerebral cortex is generated from the dorsal portion of the anterior neural tube. The signaling pathways that promote anterior neural fate and dorsal cell identities are thus likely to promote the differentiation of hESCs into cortical neurons. During early development, inhibition of the BMP signaling pathway by secreted molecules such as Noggin induce neuronal fate [18]–[20]. SB431542, a small molecule inhibitor of Smads 2, 3 in the nodal pathway [21], has been successfully used in conjunction with Noggin, referred to as dual Smad inhibition, in directing differentiating hESCs towards a dorsal neural fate [22]. During the formation of the nervous system, Sonic hedgehog (Shh) mediates the induction of ventral neurons [23], while its inhibitor, cyclopamine [24], [25], has been shown in mouse embryonic stem cells (mESCs) to increase dorsal while repressing ventral identities [26]. In human cells, however, it is not known whether cyclopamine has the same effect.

Wnt genes encode a highly conserved family of secreted glycoproteins and play an essential role in the formation of the vertebrate nervous system [27]. However, the role of the Wnt/β catenin signaling pathway in the generation of cortical neurons from hESCs is largely unknown. Conflicting reports in mESCs either claim that Wnt enhances neural differentiation [28], [29], or that it inhibits neural fate [30], [31]. Activating Wnt in mESCs has been associated with self-renewal [32]. In hESCs, the inhibition of Wnt was reported to convert hESCs-derived dorsal telencephalic progenitors to ventral progenitors [33], while its activation through GSK3β inhibition, has been reported to maintain hESCs pluripotency [34].

Overall, the specific signaling pathways promoting the generation of human subcerebral neurons including CSMNs are largely unclear. In this study, we utilized a genetically modified hESC line in which a YFP reporter was targeted into the endogenous *hFezf2* locus to investigate the differentiation of hESCs into cortical projection neurons [35]. We demonstrate that hESCs can differentiate into corticofugal neurons, including CSMNs and corticothalamic neurons *in vitro*. Upon transplantation into neonatal mouse brains, the differentiated human cells can extend axons toward the spinal cord. Our *in vitro* hESC differentiation reveals two distinct h*Fezf2*-YFP^+^ subpopulations, similar to the *Fezf2-*expressing populations in the developing mouse cortex. Interestingly, these two cell populations are molecularly analogous to mouse corticothalamic and subcerebral neurons. Overall, our study show that hESCs-derived cortical projection neurons can be used as an effective model system to investigate the molecular pathways that regulate human CSMN differentiation, axon extension and survival.

## Materials and Methods

All the human embryonic stem cell experiments and animal studies were performed in accordance with protocols approved by the IRB committees and IACUC at the University of California, Santa Cruz and performed in accordance with institutional and federal guidelines.

### Reverse Transcriptase-Polymerase Chain Reaction (RT-PCR) and Quantitative Polymerase Chain Reaction (qRT-PCR)

RNA was extracted using RNeasy (Qiagen) kit. RNA quality was evaluated using Agilent 2100 Bioanalyzer (Agilent technologies). The RNA samples were reverse-transcribed into cDNA using SuperScript III first strand synthesis system (Life Technologies). Quantitative PCR (qPCR) was performed using the ViiA 7 real-time PCR (Applied Biosystems). Expression values (CT values) were normalized with two ubiquitously expressed endogenous reference genes, Beta-2-microglobulin (B2M) and Beta-actin. The normalized expression levels of the genes in the h*Fezf2*-YFP^Hi^ and h*Fezf2*-YFP^Low^ cells were compared to those of the h*Fezf2*-YFP^Neg^ cells according to the ΔΔCT method [36]. Each sample was run in triplicate and at least three biological replicates were performed for each gene. The primers for RT-PCR and qRT-PCR are listed in Table S1 and Table S2.

### Stem Cell Culture

H9 (WA-09, passages 28 to 50) (Wicell Research Institute) cells were maintained on a feeder layer of mitotically inactivated mouse embryonic fibroblasts (MEFs) as described [37]. MEFs were plated onto coated growth factor reduced Matrigel plates (BD Biosciences) diluted 1∶20 into DMEM-F12 medium (Life Technologies). H9 were cultured in Dulbecco’s modified Eagle’s medium-F12 (DMEM-F12) supplemented with 20% Knockout serum replacement, 0.1 mM MEM nonessential amino acids (MEM NEAA), 2 mM GlutaMAX, 0.55 mM, 2-mercaptoethanol (all from Life Technologies) and 8 ng/ml of human recombinant basic fibroblast growth factor (Fgf2) (Peprotech). Cells were fed daily and passaged every 4 days with 200 units/ml of collagenase IV (Life Technologies).

The h*Fezf2-YFP* HUES-9 cells were generously provided by Drs. Binhai Zheng and Katherine Ruby from the University of California at San Diego, and used between 46–52 passages [35]. Both the h*Fezf2-YFP* HUES-9 cells and the HUES-5 cells (passages 19 to 30) (Harvard University) were cultured in knockout Dulbecco’s modified Eagle’s medium (DMEM; Life Technologies) supplemented with 10% Knockout Serum Replacement, 10% Plasmanate (Bayer HealthCare), 0.1 mM MEM nonessential amino acids, 2 mM GlutaMAX, 0.55 mM 2-mercaptoethanol (all from Life Technologies) and 15 ng/ml human basic Fgf added fresh to the medium daily. Cells were passaged every 4 days by trypsinization with 0.5% trypsin/EDTA (Life Technologies) or digestion with collagenase IV at 200 units/ml (Life Technologies) for embryoid body (EB) formation.

### Neural Differentiation from hESCs

Embryoid bodies (EBs) were formed by gentle dissociation of undifferentiated hESCs from mouse feeder layer using 200 ug/ml of collagenase IV (Life Technologies). Dissociated cells were washed three times with hESC media and were allowed to settle by gravity to form EBs in N2 medium in an ultra low attachment plate overnight (Day 0). The next day (Day 1) different growth factors or their inhibitors: SB431542 (10 um) (Sigma-Aldrich), recombinant Human Noggin (500 ng/ml), recombinant human Wnt3a (200 ng/ml) (both from R&D Systems), recombinant human DKK1 (250 ng/ml) (Peprotech), Cyclopamine (4 uM) (Biomol) were added to the N2 media. Feeding with growth factors was performed daily with a no growth factor condition used as a control. N2 media was composed of Dulbecco’s modified Eagle’s medium-F12 (DMEM-F12) medium supplemented with 0.1 mM MEM nonessential amino acids (MEM NEAA), 2 mM GlutaMAX, 1× N2 supplement (all from Life Technologies), Heparin (1 mg/ml)(Sigma-Aldrich) and human basic Fgf (10 ng/ml)(Peprotech). EBs remained for 6 days in suspension and were plated down on day 7 onto a growth factor reduced Matrigel coated plate (BD Biosciences). Feedings continued every day or every other day for 12 days. On day 12 cells were scraped from their support, resuspended in N2 media and plated down onto a new growth factor reduced Matrigel (BD Biosciences) coated plate. The next day, N2 media was replaced with freshly made differentiation media. The differentiation media was composed of Neurobasal medium supplemented with 2 mM GlutaMAX, 1× N2 supplement, 1× B-27 supplement minus vitamin A (all from Life Technologies), cAMP (1 uM), L-ascorbic acid (100 uM) (both from Sigma-Aldrich), and Human Recombinant Brain-Derived Neurotrophic Factor (BDNF) (10 ng/ml), Human Recombinant Glial-Derived Neurotrophic Factor (GDNF) (10 ng/ml) and Insulin-like Growth Factor-I (IGF-I) (10 ng/ml) (all from Peprotech). During days 13–32, cells were fed daily or every other day with differentiation medium to induce differentiation. Differentiated cells were passaged on day 21 onto a growth factor reduced Matrigel (BD Biosciences) coated plates at 1∶20 dilution and fed as described above.

### Flow Cytometry and Cell Sorting

Cells were gently dissociated using 0.5% Trypsin/EDTA (Invitrogen) followed by a neutralization step with hESC culture media containing serum as described above. Cells were then resuspended into 1XPBS, 0.5 M EDTA and 2% FBS (all from Life Technologies). Prior to sorting, propidium iodide (1 ug/ml, Molecular Probes, Invitrogen) was added to each sample at 1∶5,000 dilution to distinguish between live and dead cells. Cells were sorted on a fluorescence-activated cell sorter FACS ARIA II (BD Biosciences) with a 100 µm nozzle at 22 PSA referred to as “gentle FACS” [38]. Sorted cells were collected in 500 ul of HBSS media supplemented with 20 mM glucose, 10% FBS, 1× penicillin/streptomycin (Invitrogen). Gating was performed using unstained, or negative controls that were not expected to express *Fezf2*. Data Analysis was performed using the FlowJo software (Tree Star, Inc). All experiments, including analysis and sorting were repeated at least three times.

### Immunohistochemistry

Differentiated *Fezf2*-YFP cells and H9 cells were fixed with 4% paraformaldehyde in PBS for 15 minutes, washed twice in PBS, permeabilized with 0.1% Triton X-100 in PBS for 20 minutes, and then blocked in 5% horse serum for 1 hour. Cells were incubated overnight at 4°C with primary antibodies. After 3 PBS washes, cells were incubated in the appropriate secondary antibodies for 1 hour at room temperature protected from light. Cells were washed 3 times in 1XPBS and stained with DAPI. The primary antibodies used in this study for mouse and human cells (Table S3) were the following: chicken anti-GFP (Life Technologies), rat anti-BCL11B (Abcam), rabbit anti-TBR1 (Abcam), rabbit anti-NFIA (Active Motif), rabbit anti-NFIB (Active Motif), mouse anti-HuNu (Millipore), mouse Tuj1 antibody (Covance), rabbit anti-TBR2 (Abcam), rabbit anti-PAX6 (Covance), mouse anti-Nestin (Millipore). Secondary antibodies were as follows: Alexafluor 488, 594 and 647 (Life Technologies).

### Analysis of *Fezf2*-GFP Mouse Cells

The brains of *Fezf2*-GFP BAC transgenic mice (GENSAT) were dissected at P0 (n = 3) and immediately dissociated using Papain dissociation system (Worthington Biochemical Corporation). GFP positive and negative populations were sorted using a FACS ARIA II (BD Biosciences). 50,000–75,000 cells were sorted and processed for RNA isolation using RNeasy Plus mini kit (Qiagen) and the quality assessed using a Bioanalyzer 2100 (Agilent Technologies). cDNA was synthesized using Superscript III reverse transcriptase (Invitrogen).

### *Fezf2 In situ* Hybridization

The *in situ* hybridization using *Fezf2* probe was performed as described [39].

### Transplantation

Neural rosettes from day 12–20 were mechanically isolated from *in vitro* differentiated h*Fezf2*-YFP cells, dissociated using 0.5% Trypsin/EDTA (Invitrogen) and resuspended in serum containing media. Dissociated cells were transplanted (500–2,000 cells) using ultrasound guided imaging (VisualSonics) targeting the deep layers of the mouse motor cortex. Transplants were performed using postnatal day 0 (P0) CD-1 wild-type mice (n = 40) (Charles River Laboratories, Hollister, CA). Mice were sacrificed at P90, intracardiac perfusion were performed with fresh 4% PFA and embedded in O.C.T compound (Sakura Finetek) and kept at −80°C until sectioning was performed using a Microme sliding microtome (ThermoScientific).

## Results

### Differentiation of hESCs into Cortical Projection Neurons

**Figure 1:**
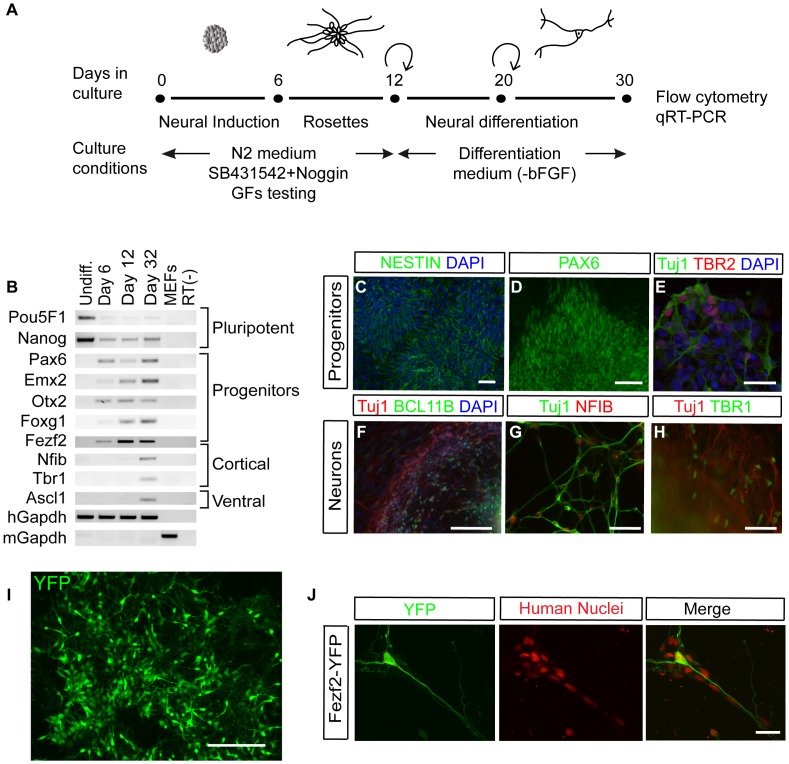
In vitro hESC neural differentiation yields cortical neurons. (**A**) Schematic representation of the strategy for differentiating hESCs into cortical neurons, displaying culture and treatments conditions. (**B**) RT-PCR of H9 cells shows differential expressions of pluripotent markers (*Pou5F1*, *Nanog*), dorsal progenitors (*Pax6, Emx2, Otx2, Foxg1*), cortical neurons (*Fezf2, Nfib, Tbr1*), and ventral marker (*Ascl1*) at neural differentiation day-6, 12 and 32. (**C–E**) Immunofluorescence staining of differentiated H9 cells shows expression of neural progenitor markers NESTIN, PAX6, TBR2 and immature neuronal marker TUJ1. (**F–H**) Immunohistochemistry shows that differentiated H9 cells express cortical neuron marker BCL11B (F), NFIB (G) and TBR1 (H). Scale bars in C, D, 50 µm; E, 25 µm; F–H, 100 µm. (**I**) Immunofluorescence staining of differentiated *Fezf2*-YFP-HUES 9 reporter line on day 40 with GFP antibody. Scale bar, 200 µm. (**J**) Differentiated *Fezf2*-YFP reporter line expressing GFP and the human specific nuclear antigen. Scale bar, 25 µm. Abbreviations: DAPI, 4′,6′-diamidino-2-phynylindole; YFP, yellow fluorescent protein; GFs, growth factors;

To generate cortical neurons from hESCs, we developed a differentiation protocol based on both current knowledge of early cortical development in mice and established hESC neural differentiation approaches [22], [40]. A schematic of our neural differentiation strategy is shown in Figure 1A, and involves three steps spanning 30 days. First, neural induction was initiated by the generation of embryoid bodies (EBs), in which undifferentiated H9 or HUES5 cells were allowed to aggregate in suspension culture, mimicking the spatial organization of the morula stage of development. Then, neural rosette formation was initiated by plating down EBs onto matrigel, supplemented with SB431542 and Noggin [20], [22], [41] for the first 12 days of culture. In the final neural differentiation stage, cells were grown in a culture medium that promoted cell survival and allowed neuronal differentiation.

Expression of forebrain progenitor markers *Pax6*, *Emx2*, *Nestin*, *Otx2*, *Foxg1*, *Tbr2* and *Fezf2* [42]–[45] was confirmed using reverse transcriptase-polymerase chain reaction (RT-PCR) (Figure 1B) and immunohistochemical staining (Figure 1C–E). *Pax6*, *Emx2* and *Foxg1* showed a progressive increase in expression throughout differentiation until day 32 as assayed by RT-PCR (Figure 1B). The intermediate progenitor marker *Tbr2* was expressed concurrently with the immature neuronal marker βIII tubulin (Tuj1) as determined by immunohistochemistry staining (Figure 1E). Subcortical neuron markers *Fezf2* and *Nfib* [*46*], corticothalamic neuron marker *Tbr1*, and CSMN marker *Bcl11b* were robustly expressed at day 32 (Figure 1B, F–H). Our neural differentiation protocol did not solely generate cortical neurons, as the expression of ventral cell marker *Ascl1* [47] was detected at day 32 (Figure 1B). Expression of the pluripotent marker *Nanog* [48] was also maintained throughout the neuronal differentiation procedure, suggesting that some cells retained their stem cell characteristics throughout differentiation (Figure 1B). However, another pluripotency gene, *Pou5F1* [49] (also known as *Oct4*) showed significant down-regulation upon differentiation (Figure 1B).

**Figure 2:**
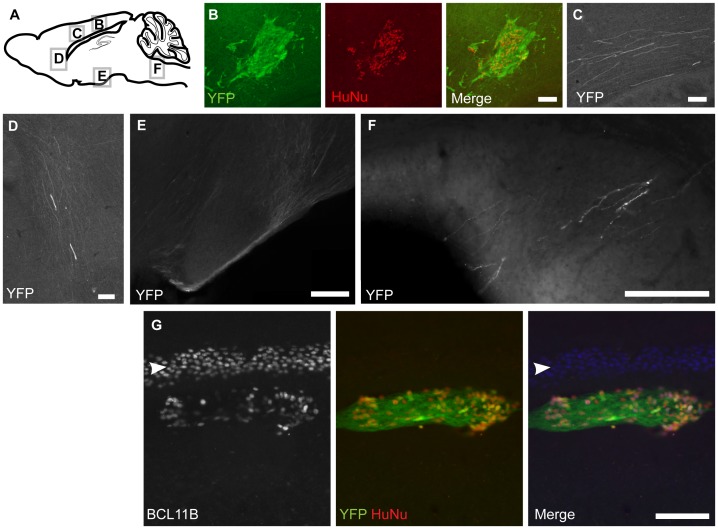
Human ESCs-derived *Fezf2-*YFP-expressing neurons extend axonal projections in the mouse brain at P90. (**A**) Schematic of sagittal mouse brain indicates the location of panels B–F. YFP^+^HuNu^+^ cells were present in the injection site in the cortex (**B**), some YFP+ axons were present in the white matter (**C, D**), at the base of the forebrain (**E**), and pyramidal decussation (**F**). (**G**) Transplanted h*Fezf2*-YFP cells at the injection site in the deep layer of the cortex expressed the CSMN marker BCL11B. Mouse layer 5 neurons expressing BCL11B are indicated by white arrow. Scale bars: 100 µm (B, C, F, G); 200 µm (D, E).

*Fezf2* is a specific marker for corticofugal neurons in mouse and human [16], [17]. To further investigate the hESC-derived subcerebral neurons, we differentiated a genetically modified hESC line (*Fezf2*-YFP HUES9) in which the YFP reporter was inserted into the endogenous human *Fezf2* gene locus [35]. Following our neuronal differentiation protocol, many *Fezf2*-YFP*-*expressing cells extended long neuronal processes by day 40 (Figure 1 I, J and Figure 2). In addition to H9 (Figure 1B–H) and *Fezf2*-YFP HUES9 (Figure 1I, J) lines, our neuronal differentiation protocol also successfully generated cortical projection neurons, including CSMNs, from HUES 5 cells (Figure S1).

### Transplanted hESCs-derived *Fezf2*-YFP-expressing Neurons Extend Axons in the Mouse Brain

To determine whether *in vitro* differentiated corticofugal neurons could integrate and project their axons to appropriate targets, we tested their ability to extend axons subcortically upon transplantation into neonatal mouse cortices. We dissociated neural rosettes from day 12 cultures and injected them using ultra-sound guided imaging into the P0 cortex of wild-type mice. Ninety days after transplantation, the recipient mice were sacrificed and their brains analyzed with GFP and human nuclei (HuNu) antibodies by immunohistochemistry (Figure 2). The expression of the human nuclear antigen confirmed the human origin of the transplanted cells (Figure 2B). Consistent with corticofugal identity, many *Fezf2*-YFP^+^ cells were observed extending axons from the cell injection site in the cortex to subcortical targets (Figure 2A). *Fezf2*-YFP^+^ axons were observed in the white matter (Figure 2C), and turning into the internal capsule (Figure 2D). YFP^+^ axons were also observed at the base of the ventral forebrain (Figure 2E), and in the pyramidal decussation on their way to the spinal cord (Figure 2F). Injected *Fezf2*-YFP cells in the cortex expressed the CSMN marker BCL11B (Figure 2G). These experiments demonstrated that transplanted hCSMNs integrated into the host brains and extended axons toward subcortical targets, similar to endogenous *Fezf2-*expressing projection neurons.

### Differentiated hESCs Contain Two Distinct *Fezf2*-YFP^+^ Sub-populations

**Figure 3:**
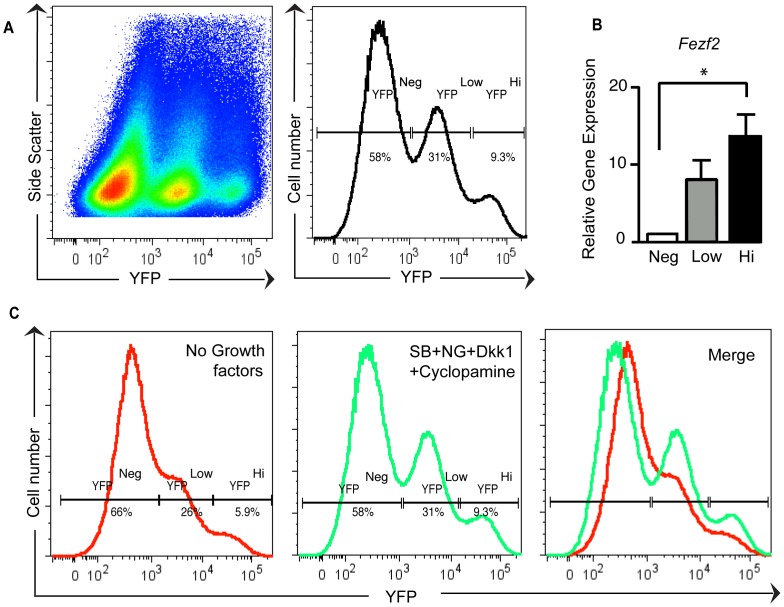
Differentiated hESCs contain two distinct *hFezf2*-YFP^+^ sub-populations detected by FACS under different cell culture conditions. (**A**) Representative FACS pseudo-color plot and FACS histogram of differentiated *Fezf2*-YFP cells on day 30, showing three subpopulations based on h*Fezf2*-YFP expression: YFP^Neg^, YFP^Low^, and YFP^Hi^. (**B**) Quantitative RT-PCR of h*Fezf2* mRNA from the three sorted cell subpopulations (F(2,6) = 8.655; p = 0.0171; n = 3). (**C**) Representative FACS histograms of differentiated h*Fezf2*-YFP cells under no growth factors treatment and h*Fezf2*-YFP cells from the same experiment treated with SB+NG+DKK1+cyclopamine. (p and F values were assessed by one-way ANOVA with Tukey’s post-test.) Abbreviations: SB, SB431542; NG, Noggin; DKK1, Dickkopf-1.

To identify the culture conditions that efficiently promote hESC differentiation into CSMNs, we differentiated *Fezf2*-YFP HUES9 cells under different growth factor conditions. We subsequently performed Fluorescence Activated Cell Sorting (FACS) analysis at day 30 of differentiation to quantify the percentage of *Fezf2*-expressing cells. Our goal was to identify the culture conditions most efficient at promoting the generation of *Fezf2*-YFP-expressing neurons. Treatment with SB431542, Noggin, DKK1 and cyclopamine, as described in Figure 1, resulted in an increase in the percentage of *Fezf2*-expressing cells as compared to the no growth factor control (Figure 3C). Interestingly, under these different differentiation conditions, in addition to a *Fezf2*-YFP^−^ population, we consistently observed two distinct *Fezf2*-YFP^+^ cell populations (Figure 3A,C): cells expressing high levels of *Fezf2*-YFP (YFP^Hi)^ and cells expressing low levels of *Fezf2*-YFP (YFP^Low)^ (Figure. 3A). To confirm the presence of these *Fezf2-*YFP^+^ populations, we performed qRT-PCR on each sorted population to test the expression levels of the human *Fezf2* transcript. As expected, *Fezf2* was highly expressed in the YFP^Hi^ sorted subpopulation compared to the *Fezf2*-YFP^−^ cell population F(2,6) = 8.655; p = 0.0171; n = 3) (Figure 3B), whereas expression of *Fezf2* in the YFP^Low^ population was intermediate between *Fezf2*-YFP^−^ and *Fezf2*-YFP^Hi^ populations (Figure. 3B). Interestingly, both YFP^Hi^ and YFP^Low^ populations were observed even when differentiation occurred in the absence of growth factors (Figure 3C, first panel). Thus, our *Fezf2*-YFP HUES9 neural differentiation protocol generated two novel and distinct *Fezf2*-YFP^+^ populations (Figure 3A,C), suggesting they might exhibit different molecular characteristics.

### Inhibiting Wnt Signaling Promotes the Generation of *Fezf2*-YFP-expressing Cells

**Figure 4:**
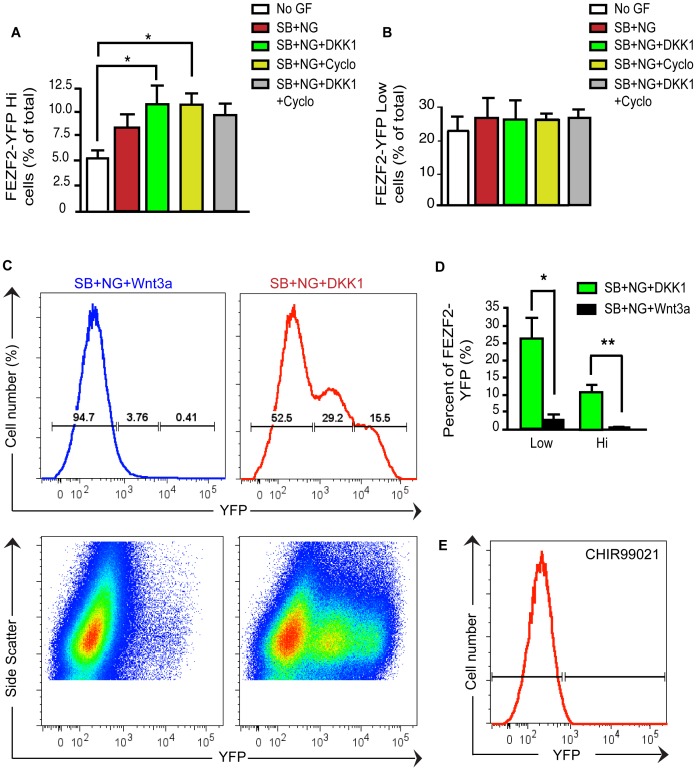
TGFß/BMP/Wnt-Shh triple inhibition activates *Fezf2* expression in hESCs. (**A**) Quantification by FACS of differentiated h*Fezf2*-YFP cells under different conditions shows a 2.2 fold increase in efficiencies in generating h*Fezf2*-YFP^Hi^ subpopulation using SB+NG+DKK1 and SB+NG+Cyclopamine treatments compared to a no growth factor control F(4,20) = 13.74) p = 0.0235. Error bars represent standard error of mean, sem (n = 4). p and F values were assessed by one-way ANOVA with Tukey’s post-test). The efficiency of generating *Fezf2*-GFP^Hi^ cells was in the range: 5–10.8%, n = 4 per condition, quantified by FACS. (**B**) FACS analysis of differentiated h*Fezf2*-YFP cells shows that the efficiencies to generate h*Fezf2*-YFP^Low^ subpopulation were similar under different conditions (range: 23–26.7%, n = 4) but yielded an overall higher percentage than the *Fezf2*-GFP^Hi^ population. (**C**) Representative FACS histograms and FACS dot plots showing differentiated h*Fezf2*-YFP cells under Wnt activation (Wnt3a) and Wnt inhibition (DKK1). (**D**) Quantification of the efficiencies of generating h*Fezf2*-YFP^Hi^ and h*Fezf2-*YFP^Low^ subpopulations under Wnt activation and Wnt inhibition conditions. Student t-test, unpaired, two-tailed, Welch’s corrected t = 3.91; p = 0.0298 (for low) and t = 6.329; p = 0.0080 (for high). (**E**) FACS histogram showing inhibition of both h*Fezf2*-YFP^Hi^ and h*Fezf2-*YFP^Low^ populations under treatment with the Wnt agonist CHIR99021 at day 30.

Next we sought to determine whether activating or inhibiting specific signaling pathways during the first 12 days of the neural differentiation protocol affected the efficiency of generating *Fezf2*-YFP^+^ cells. Interestingly, the frequency of *Fezf2*-YFP^Low^ cells (Figure 4B) on day 30 was consistently higher (range: 23–26.7%, n = 4 per differentiation condition) than the frequency of *Fezf2*-YFP^Hi^ cells (range: 5–10.8%, n = 4 per condition) (Figure 4A). In addition, *Fezf2*-YFP^Hi^ and *Fezf2*-YFP^Low^ subpopulations responded differently to the 5 treatments conditions tested, which included 1) no growth factor, 2) dual SMAD inhibition, 3) dual SMAD inhibition plus Dickkopf-1 (DKK1, inhibiting Wnt signaling pathway), 4) dual SMAD inhibition plus cyclopamine (inhibiting Shh pathway), and 5) dual SMAD inhibition plus DKK1 and cyclopamine.

The generation of *Fezf2*-YFP^Hi^ cells was more sensitive to inhibition from the TGFß/BMP/Wnt-Shh signaling pathways as compared to the *Fezf2*-YFP^Low^ population (Figure 4A). Inhibition of the Wnt pathway using the secreted protein DKK1 induced a 2-fold increase in the *Fezf2*-YFP^Hi^ subpopulation over the no growth factor control (Figure 4A). A similar increase in induction of the *Fezf2*-YFP^Hi^ subpopulation was observed upon inhibition of Shh signaling with cyclopamine. However, we did not observe further enhanced induction of the *Fezf2*-YFP^Hi^ population when using both DKK1 and cyclopamine, suggesting a lack of synergism between inhibiting these two pathways. In contrast to the YFP^Hi^ population, generation of YFP^Low^ cells was not significantly influenced by inhibition of TGFß/BMP, Shh or Wnt signaling pathways (Figure 4B). Despite the ability to induce YFP^Hi^ cells by growth factor treatment, the percentage of YFP^Hi^ expressing cells was consistently lower compared to the YFP^Low^ cells. The difference in response to inhibition of signaling pathways, as well as the difference in population sizes suggested that *Fezf2*-YFP^Low^ and *Fezf2*-YFP^Hi^ cells represented two distinct populations.

To further determine the effects of activating and inhibiting Wnt signaling on generating *Fezf2*-YFP-expressing cells from hESCs, we next assessed the effect of Wnt activation by addition of Wnt3a to the media. We observed an almost complete loss of both *Fezf2*-YFP^Hi^ and *Fezf2-*YFP^Low^ populations upon addition of Wnt3a (Figure 4C, D). Compared to inhibition of Wnt signaling with DKK1, the percentage of *Fezf2*-YFP^Hi^ cells at day 30 showed a 39.8 fold decrease (n = 3) and the percentage of *Fezf2*-YFP^Low^ cells showed a 10.65 fold decrease (n = 3) when Wnt3a was added to the media (Figure 4C,D). Moreover, when we substituted Wnt3a with the small molecule Wnt agonist CHIR99021, the generation of *Fezf2*-YFP-expressing cells was completely inhibited (Figure 4E). Wnt activation therefore seems to have strong inhibitory effect on the generation of both h*Fezf2* ^Hi^ and h*Fezf2* ^Low^ populations. Collectively, these experiments demonstrate that the differentiation of h*Fezf2*-YFP-expressing populations and *Fezf2* expression was robustly activated by the triple inhibition of TGFß/BMP/Wnt-Shh signaling.

### Differentiated h*Fezf2*-YFP^hi^ and h*Fezf2*-YFP^Low^ Populations Recapitulate *Fezf2* Expression during Mouse Cortical Development

**Figure 5:**
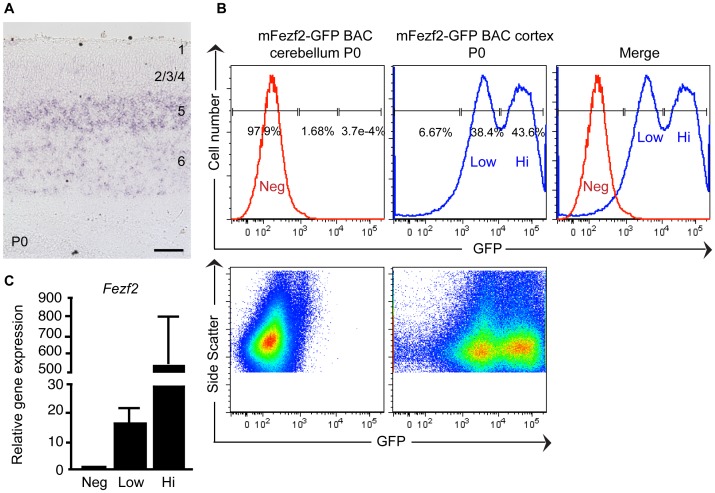
*Fezf2*-GFP BAC transgenic mice contain two distinct *Fezf2*-GFP^+^ populations. (**A**) *In situ* hybridization shows that a P0 wild-type mouse cortex contains layer 5 neurons expressing *Fezf2* at a high level and layer 6 neurons expressing *Fezf2* at a low level. (**B**) Representative FACS histograms and dot plots of P0 *Fezf2*-GFP BAC transgenic mice show that the cerebellum contains a m*Fezf2*-GFP^Neg^ population, and the cerebral cortex contains m*Fezf2*-GFP^Hi^ and m*Fezf2*-GFP^Low^ populations. (**C**) Quantitative RT-PCR analysis of m*Fezf2* mRNA expression levels in sorted m*Fezf2*-GFP^Hi^, m*Fezf2*-GFP^Low^ and mFezf2-GFP^Neg^ sub-populations.

To determine whether the *hFezf2*-YFP^Hi^ and *hFezf2*-YFP^Low^ populations arose from a cell culture artifact or were biologically relevant, we analyzed *Fezf2* expression in the developing mouse cortex to determine if these two populations were present *in vivo*. Since a FEZF2 antibody suitable for immunohistochemistry is not available, we performed *in situ* hybridization to detect *Fezf2* expression at P0 in the mouse cortex (Figure 5A). As previously reported [10], *Fezf2* mRNA expression was observed in two different cortical layers: a higher level of *Fezf2* expression was detected in layer 5, while a lower *Fezf2* expression level was detected in layer 6 (Figure 5A). To further investigate whether there were two distinct *Fezf2^Hi^* and *Fezf2^Low^* populations in the developing mouse cerebral cortex, we analyzed cell populations in the *Fezf2*-GFP BAC transgenic mouse containing a modified bacterial artificial chromosome in which an EGFP open reading frame was inserted at the start codon of the mouse *Fezf2* gene.

We dissected cortices from P0 *Fezf2*-GFP BAC mice, dissociated the cells, and analyzed GFP-expressing cells by FACS (Figure 5B). Dissociated cells from the cerebellum of *Fezf2*-GFP BAC mice, which do not express *Fezf2*, were used as the negative control. We found that two distinct *Fezf2*-GFP-expressing populations existed in the P0 mouse cortex (Figure 5B). To directly examine the expression levels of endogenous *Fezf2* mRNA in these populations, we sorted *Fezf2*-GFP^Neg^, *Fezf2*-GFP^Hi^, and *Fezf2*-GFP^Low^ populations, and performed qRT-PCR (Figure 5C). *Fezf2* was not expressed in *Fezf2*-GFP^Neg^, but was highly expressed in *Fezf2*-GFP^Hi^ population with a 33-fold increase (n = 3) over the *Fezf2-*GFP^low^ population (Figure 5C). These results demonstrated that two *Fezf2*-expressing populations existed in the developing mouse cortex and could be successfully isolated by FACS, therefore suggesting that the *Fezf2*-YFP^Hi^ and *Fezf2*-YFP^Low^ cells generated from hESCs were biologically relevant, and likely recapitulated the two populations of *Fezf2*-expressing neurons present during mammalian cortical development.

### Differentiated *Fezf2-*YFP^Hi^ and *Fezf2-*YFP^Low^ Cells Express Corticofugal Neuron Markers

**Figure 6:**
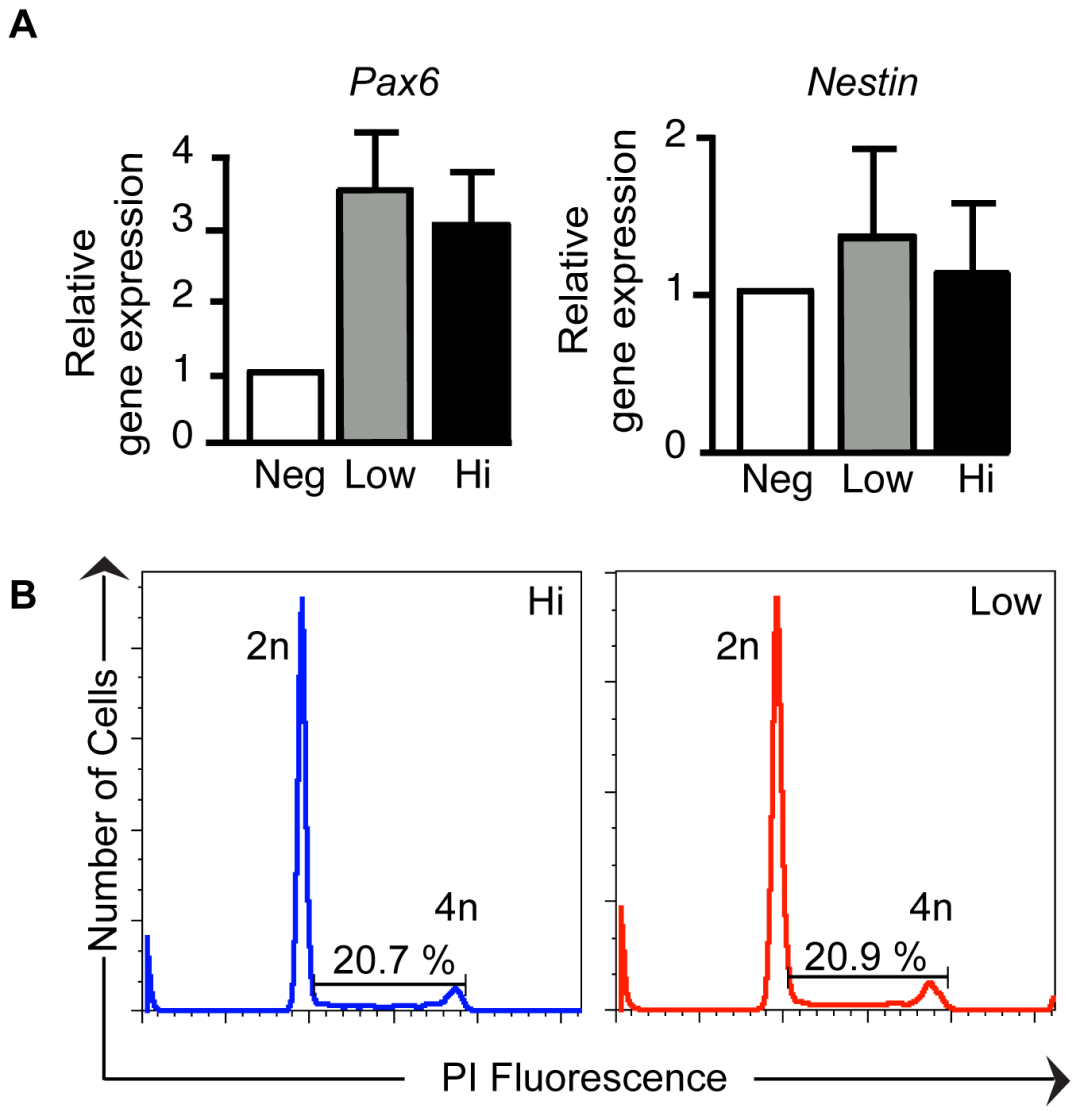
Differentiated h*Fezf2*-YFP cells at day 30 share similar cell cycle kinetics. (**A**) Quantitative RT-PCR analysis of progenitor markers, *Pax6* and *Nestin*, on differentiated and sorted cells at day 30. (**B**) Flow cytometry analysis of cell cycle properties of differentiated and sorted h*Fezf2*-YFP cells. The cells were stained with propidium iodide to reveal DNA content. Abbreviation: PI, propidium iodide.

Next, we sought to determine the molecular identities of the differentiated *Fezf2-*YFP^Hi^ and *Fezf2-*YFP^Low^ cell populations by comparing their gene expression. We first investigated whether these populations differed in their progenitor versus post-mitotic neuronal states by performing qRT-PCR analysis on the h*Fezf2*-YFP-expressing populations at day 30. Indeed, *Fezf2* is a gene expressed during early mouse development (E8.5) and continues to be expressed in post-mitotic neurons in subcortical projection neurons [11]. Expression levels of neural progenitor markers *Pax6* and *Nestin* were not significantly different between *Fezf2*-YFP^Hi^ and *Fezf2*-YFP^Low^ populations at day 30 (Figure 6A). Cell cycle analysis based on DNA content by FACS confirmed that *Fezf2*-YFP^Hi^ and *Fezf2*-YFP^Low^ cell populations shared similar cell cycle kinetics and that approximately 20% of the cells were proliferating in both populations (Figure 6B).

**Figure 7:**
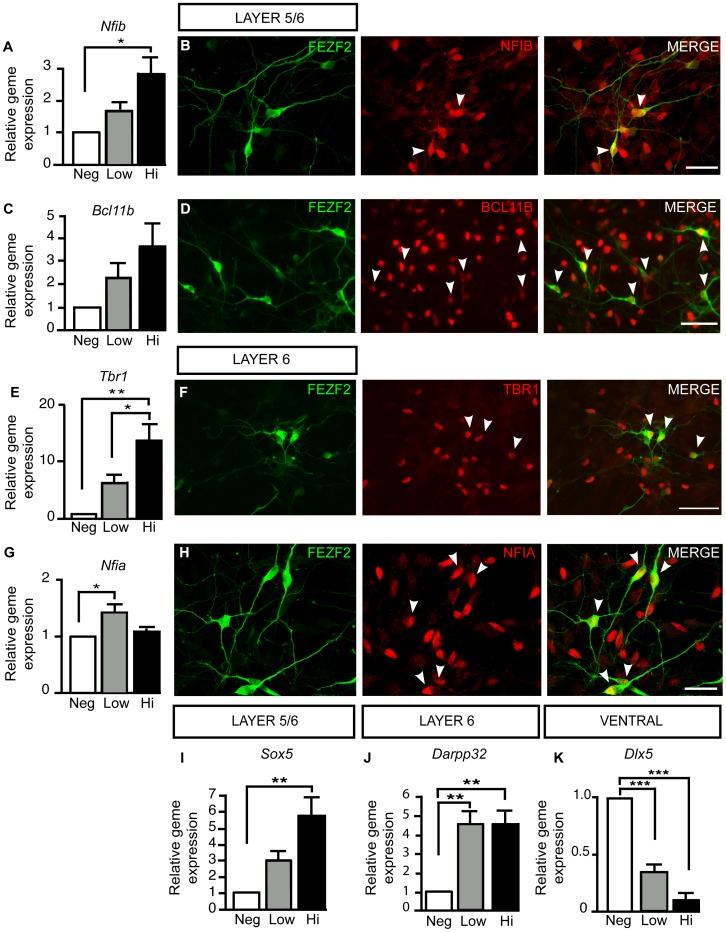
Differentiated h*Fezf2-*YFP^Hi^ and h*Fezf2-*YFP^Low^ cells express corticofugal neuron markers. (**A**) Quantitative RT-PCR analysis for *Nfib* mRNA expression on sorted h*Fezf2*-YFP subpopulations (F(2,6) = 7.405; p = 0.0240). (**B**) Immunofluorescence staining showing NFIB expression in differentiated h*Fezf2*-YFP+ cells at day 35. (**C**) Quantitative RT-PCR analysis of *Bcl11b* mRNA expression on sorted h*Fezf2*-YFP subpopulations. (**D**) Immunofluorescence staining showing BCL11B expression in differentiated h*Fezf2*-YFP+ cells at day 40. (**E**) Quantitative RT-PCR analysis of *Tbr1* mRNA expression on sorted h*Fezf2*-YFP subpopulations (F(2,6) = 15.63; p = 0.0042). (**F**) Immunofluorescence staining showing TBR1 expression in differentiated h*Fezf2*-YFP cells at day 40. (**G**) Quantitative RT-PCR expression of *Nfia* mRNA expression on sorted h*Fezf2*-YFP subpopulations. (F(2,6) = 7.316; p = 0.0246). (**H**) Immunofluorescence staining showing NFIA expression in differentiated h*Fezf2*-YFP cells at day 40. (**I**) Quantitative RT-PCR analysis of *Sox5* mRNA on sorted h*Fezf2*-YFP subpopulations. (F(2,6) = 11.87; p = 0.0082). (**J**) Quantitative RT-PCR analysis of *Darpp32* mRNA on sorted h*Fezf2*-YFP subpopulations. (F(2,6) = 14.80; p = 0.0048). (**K**) Quantitative RT-PCR analysis of *Dlx5* mRNA on sorted h*Fezf2*-YFP subpopulations. (F(2,6) = 58.90; p = 0.001). Statistical analysis: ANOVA with Tukey’s post-test.

We next investigated whether differentiated h*Fezf2*-YFP^Hi^ and h*Fezf2*-YFP^Low^ populations preferentially expressed markers for cortical projection neurons of layer 5 and/or 6, since *in situ* hybridization revealed that *Fezf2* was expressed in layers 5 and 6 of the mouse cortex, where subcerebral and corticothalamic neurons are respectively located (Figure 5A). Cortical neuron markers expressed at a high level in layer 5 subcerebral neurons, and low level in layer 6 corticothalamic neurons in mice include *Nfib*, *Bcl11b*, and *Sox5* (Figure S2), in addition to *Fezf2* (Figure 5A). When h*Fezf2*-YFP^Neg^, h*Fezf2*-YFP^Low^ and h*Fezf2*-YFP^Hi^ populations were tested for these markers by qRT-PCR, *Nfib*, *Bcl11b* and *Sox5* showed the general trend of being more highly expressed in h*Fezf2*-YFP^Hi^ compared to h*Fezf2*-YFP^Low^ cells (Figure 7A, C, I). Conversely, genes expressed at high levels in layer 6 corticothalamic neurons in mice, such as *Nfia* [*50*] (Figure S3) showed elevated levels of expression in *Fezf2*-YFP^Low^ cells as compared to *Fezf2*-YFP^Hi^ cells (Figure 7G). These results suggested that h*Fezf2* ^Hi^ cells were more similar to layer 5 subcerebral neurons whereas the h*Fezf2* ^Low^ population was more similar to layer 6 corticothalamic neurons. However, expression of another layer 6 corticothalamic marker, *Tbr1* (Figure S3), showed a highly elevated expression in the *Fezf2*-YFP^Hi^ population, with a 2 fold increase over *Fezf2*-YFP^Low^ (Figure 7E). In addition, expression levels of *Darpp32* (Figure S3) were comparable between the *Fezf2*-YFP^Low^ and the *Fezf2*-YFP^Hi^ cells (Figure 7J). These results suggested that h*Fezf2*-YFP^Hi^ population showed both layer 5 and 6 (corticospinal and corticothalamic neurons) molecular characteristics. The ventral marker *Dlx5* [*51*] was not expressed in either *Fezf2*-YFP^Hi^ or *Fezf2*-YFP^Low^ populations, confirming the enrichment of corticofugal neurons in h*Fezf2* ^+^ cell populations (Figure 7K). Immunohistochemical analysis of differentiated h*Fezf2*-YFP cells further confirmed that h*Fezf2*-YFP^+^ cells expressed NFIB, BCL11B, TBR1, and NFIA proteins (Figure 7B, D, F, H). These results suggest that *Fezf2*-YFP^Hi^ is most similar to layer 5–6 projection neurons and *Fezf2*-YFP^Low^ cells more similar to layer 6. Taken together, these data confirm that h*Fezf2-*YFP^+^ cells represent deep-layer corticofugal projection neurons.

## Discussion

Human embryonic stem cells allow the manipulation of cellular signaling pathways thus providing researchers with an *in vitro* tool to assess human cortical cell fate specification. While well studied in mouse models [4], [5], [7], [8], [10], [11], [13], [52], [53], modulation of signaling pathways that regulate projection neuron fate is challenging in human cells largely due to the lack of relevant models. Here we report the successful generation of corticofugal neurons, including corticospinal and corticothalamic neurons from human ESCs that are able to integrate upon transplantation in a manner suggesting corticofugal identity.

Our study identified two distinct *Fezf2* subpopulations in differentiated hESCs that are reminiscent of *Fezf2-*expressing subpopulations in the mouse cortex. The two *Fezf2-*expressing cell populations isolated in this study demonstrate that *Fezf2-*expressing cells exist in phenotypically distinct states, with one subpopulation expressing layer 5, 6 markers, and the other expressing layer 6 markers. While the *Fezf2* ^Hi^ population, predominantly expressed layer 5 markers, the corticothalamic marker (layer 6) *Tbr1* was also highly expressed in this population. In addition, *Darpp32*, a gene highly expressed in layer 6 neurons in mouse (Figure S3), showed comparable expression levels between the human *Fezf2*-YFP^Low^ and *Fezf2*-YFP^Hi^ populations. These results indicate that the *Fezf2*-YFP^Hi^ population was not strictly analogous to CSMN (layer 5), but instead exhibited molecular characteristics of deep layer projection neurons in general. It is also possible that human subcortical neurons are different from the mouse subcortical neurons, and that the different gene expression levels observed between the mouse subcerebral and corticothalamic neurons may not be identical in human subcortical projection neurons. We also investigated the formation of the two *Fezf2-*YFP*^+^* populations after day 30 and observed whether a potential conversion of *Fezf2*-YFP^Low^ to a *Fezf2*-YFP^Hi^ population was occurring. Our data showed that *Fezf2* subpopulations were still clearly distinct after day 100 (data not shown), suggesting that Fezf2-YFP^Low^ is not a precursor of *Fezf2*-YFP^Hi^ but instead a *bona fide* population. However, since the human ES cell differentiation procedure did not fully recapitulate the *in vivo* brain development condition, it is possible that even though we were able to generate neurons with subcortical neuron features, chromatin state and gene expression levels in the differentiated neurons were not identical to endogenous human subcortical neurons. Our findings highlight the use of the hESC system not only to dissect signaling pathways affecting neuronal differentiation, but also to identify cell heterogeneity within a population of differentiated neurons. Further delineation of the characteristics of these two populations by transplant experiments, which have thus far been difficult to execute due to low live cell recovery rate after FACS, would provide more information on their heterogeneity and distinct functions.

Previous studies have established a role for TGFß/BMP inhibition using the small molecule SB431542 and the BMP inhibitor Noggin in the differentiation of hESCs towards a neuroectodermal fate [22], [54]. Motor neurons have been differentiated from induced pluripotent stem cells (iPS) or hESCs using Shh agonists and retinoic acid [55]–[58]. Recently, the retinoid-signaling pathway induced by vitamin A treatment was shown to efficiently generate cortical neurons from hESCs and iPS cells [59]. Using h*Fezf2*-YFP as a marker, we investigated the effects of different signaling pathways on generating corticofugal neurons. Our study shows that the inhibition of both Wnt and Shh pathways enriches for h*Fezf2*-YFP-expressing neurons. Landmark studies in zebrafish and mouse model systems have established that Wnt antagonism is required for proper telencephalic patterning early in development [60]–[63]. *Fezf2* is expressed from E8.5 in early mouse development and functions in the rostro-caudal (anterio-posterior or A/P) patterning of the forebrain [64]. The expression of *Fezf2* early in development and its function in A/P axis places this gene at the crossroads of important patterning events involving the Wnt pathway, and could explain the complete repression of h*Fezf2*-YFP by Wnt *in vitro*. Incidentally, *Fezf2* was first identified in a DKK1 overexpression screen in zebrafish, and showed low or no expression in embryos overexpressing Wnt8b [65]. This study further demonstrated that *Fezf2* overexpression in zebrafish inhibited Wnt1 expression. A separate study showed that *Fezf2* was able to rescue a headless (*hdl/tcf3)* mutant embryo, encoding a Wnt transcriptional repressor [66]. Overall, these studies suggest that Wnt and *Fezf2* act in a common pathway and that Wnt signaling might act upstream of *Fezf2* [64]. Interestingly, in Xenopus, the co-expression of BMP and Wnt inhibitors cooperate to induce ectopic head structures [62], suggesting a “two-inhibitor model” of anterior-posterior (A/P) neural induction, in which BMP inhibition alone induces posterior neural patterning while BMP-Wnt inhibition anteriorizes neural structures [67], [68]. This model could extend to our finding that triple inhibition of TGFß/BMP/Wnt-Shh signaling induces h*Fezf2* expression in hESCs.

### Conclusions

Overall this study demonstrates that hESCs are a useful model system to dissect signaling pathways functioning during neuronal differentiation towards corticofugal projection neurons. We demonstrate that triple inhibition of TGFß/BMP/Wnt-Shh enhances the differentiation of h*Fezf2*-expressing cells greater than 2-fold over no growth factor control condition. Furthermore, we uncover the presence of two novel and distinct h*Fezf2-*YFP-expressing subpopulations suggesting that *Fezf2*-expressing cells are heterogenous. Knowledge derived from such studies can be used for *in vitro* differentiation of patient-derived induced pluripotent stem cells (iPSCs) into corticospinal motor neurons, elucidate disease mechanisms underlying upper motor neuron diseases, as well as provide a platform to discover potential targets for drug development.

## Supporting Information

Figure S1 **Reverse Transcriptase PCR (RT-PCR) shows expression of cortical cell markers in differentiated HUES 5 cells.** Pluripotent markers *Pou5F1* and *Nanog* are expressed in undifferentiated cells, their expression persists throughout differentiation albeit at lower levels. Radial glia marker, *Pax6* is strongly expressed at day 6 and can still be detected at day 60. The neural progenitor marker *Emx2* is detected starting at day 6 and its expression increases throughout differentiation until day 60. The corticospinal motor neuron *Fezf2* is first detected at day 6 and is strongly expressed at day 60. Corticofugal neuron markers *Bcl11b, Nfib* and *Tbr1* are all robustly expressed at day 60. None of the markers show expression in mouse embryonic fibroblasts (MEFs) control.
(TIF)

Figure S2 **Immunohistochemistry of wild-type mouse cortex showing corticofugal neuron marker expression at P0. (A)** Subcortical neuron marker BCL11B (green) is expressed strongly in layer 5 and weakly in layer 6 of the mouse cortex. DAPI indicates nuclear staining (blue) in all sections. **(B)** Corticofugal neuron marker SOX5 (green) is expressed in layer 5–6 of the mouse cortex. **(C)** Subcortical neuron marker NFIB (green) is expressed in layer 5–6 of the mouse cortex. Abbreviations: DAPI, 4′,6-diamidino-2-phenylindole; Scale bars: 150 µm.
(TIF)

Figure S3 **Immunohistochemistry of wild-type mouse cortex showing expression of layer 6 markers at P0. (A)** NFIA (green) is expressed strongly in layer 6 of the mouse cortex. DAPI indicates nuclear staining (blue) in all sections. **(B)** DARPP32 (green) is strongly expressed in layer 6 of the mouse cortex. **(C)** Corticothalamic marker TBR1 (green) is expressed strongly in layer 6. Abbreviations: DAPI, 4′,6-diamidino-2-phenylindole; Scale bars: 150 µm.
(TIF)

Table S1 **Human and mouse RT-PCR primers.**
(DOCX)

Table S2 (DOCX)

Table S3 **Primary Antibodies used in this study.**
(DOCX)

## References

[1] Bruijn LI, Miller TM, Cleveland DW (2004) Unraveling the mechanisms involved in motor neuron degeneration in ALS. Annu Rev Neurosci 27: 723–749. 15217349 10.1146/annurev.neuro.27.070203.144244

[2] Pasinelli P, Brown RH (2006) Molecular biology of amyotrophic lateral sclerosis: insights from genetics. Nat Rev Neurosci 7: 710–723. 16924260 10.1038/nrn1971

[3] Arlotta P, Molyneaux BJ, Jabaudon D, et al (2008) Ctip2 controls the differentiation of medium spiny neurons and the establishment of the cellular architecture of the striatum. J Neurosci 28: 622–632. 18199763 10.1523/JNEUROSCI.2986-07.2008 PMC6670353

[4] Chen B, Wang SS, Hattox AM, et al (2008) The Fezf2-Ctip2 genetic pathway regulates the fate choice of subcortical projection neurons in the developing cerebral cortex. Proc Natl Acad Sci U S A 105: 11382–11387. 18678899 10.1073/pnas.0804918105 PMC2495013

[5] Bedogni F, Hodge RD, Elsen GE, et al. Tbr1 regulates regional and laminar identity of postmitotic neurons in developing neocortex. Proc Natl Acad Sci U S A 107: 13129–13134. 10.1073/pnas.1002285107 PMC2919950 20615956

[6] McKenna WL, Betancourt J, Larkin KA, et al (2011) Tbr1 and Fezf2 regulate alternate corticofugal neuronal identities during neocortical development. J Neurosci 31: 549–564. 21228164 10.1523/JNEUROSCI.4131-10.2011 PMC3276402

[7] Han W, Kwan KY, Shim S, et al (2011) TBR1 directly represses Fezf2 to control the laminar origin and development of the corticospinal tract. Proc Natl Acad Sci U S A 108: 3041–3046. 21285371 10.1073/pnas.1016723108 PMC3041103

[8] Kwan KY, Lam MM, Krsnik Z, et al (2008) SOX5 postmitotically regulates migration, postmigratory differentiation, and projections of subplate and deep-layer neocortical neurons. Proc Natl Acad Sci U S A 105: 16021–16026. 18840685 10.1073/pnas.0806791105 PMC2572944

[9] Joshi PS, Molyneaux BJ, Feng L, et al (2008) Bhlhb5 regulates the postmitotic acquisition of area identities in layers II–V of the developing neocortex. Neuron 60: 258–272. 18957218 10.1016/j.neuron.2008.08.006 PMC2643370

[10] Molyneaux BJ, Arlotta P, Hirata T, et al (2005) Fezl is required for the birth and specification of corticospinal motor neurons. Neuron 47: 817–831. 16157277 10.1016/j.neuron.2005.08.030

[11] Chen B, Schaevitz LR, McConnell SK (2005) Fezl regulates the differentiation and axon targeting of layer 5 subcortical projection neurons in cerebral cortex. Proc Natl Acad Sci U S A 102: 17184–17189. 16284245 10.1073/pnas.0508732102 PMC1282569

[12] Chen JG, Rasin MR, Kwan KY, et al (2005) Zfp312 is required for subcortical axonal projections and dendritic morphology of deep-layer pyramidal neurons of the cerebral cortex. Proc Natl Acad Sci U S A 102: 17792–17797. 16314561 10.1073/pnas.0509032102 PMC1308928

[13] McKenna WL, Betancourt J, Larkin KA, et al (2011) Tbr1 and Fezf2 regulate alternate corticofugal neuronal identities during neocortical development. J Neurosci 31: 549–564. 21228164 10.1523/JNEUROSCI.4131-10.2011 PMC3276402

[14] Rouaux C, Arlotta P (2010) Fezf2 directs the differentiation of corticofugal neurons from striatal progenitors in vivo. Nat Neurosci 13: 1345–1347. 20953195 10.1038/nn.2658 PMC4207442

[15] Franco SJ, Muller U (2013) Shaping our minds: stem and progenitor cell diversity in the Mammalian neocortex. Neuron 77: 19–34. 23312513 10.1016/j.neuron.2012.12.022 PMC3557841

[16] Zhu H, Yang Y, Gao J, et al (2010) Area dependent expression of ZNF312 in human fetal cerebral cortex. Neurosci Res 68: 73–76. 20570630 10.1016/j.neures.2010.05.007

[17] Johnson MB, Kawasawa YI, Mason CE, et al (2009) Functional and evolutionary insights into human brain development through global transcriptome analysis. Neuron 62: 494–509. 19477152 10.1016/j.neuron.2009.03.027 PMC2739738

[18] Smith WC, Harland RM (1992) Expression cloning of noggin, a new dorsalizing factor localized to the Spemann organizer in Xenopus embryos. Cell 70: 829–840. 1339313 10.1016/0092-8674(92)90316-5

[19] Wilson PA, Hemmati-Brivanlou A (1995) Induction of epidermis and inhibition of neural fate by Bmp-4. Nature 376: 331–333. 7630398 10.1038/376331a0

[20] Lamb TM, Knecht AK, Smith WC, et al (1993) Neural induction by the secreted polypeptide noggin. Science 262: 713–718. 8235591 10.1126/science.8235591

[21] Inman GJ, Nicolas FJ, Callahan JF, et al (2002) SB-431542 is a potent and specific inhibitor of transforming growth factor-beta superfamily type I activin receptor-like kinase (ALK) receptors ALK4, ALK5, and ALK7. Mol Pharmacol 62: 65–74. 12065756 10.1124/mol.62.1.65

[22] Chambers SM, Fasano CA, Papapetrou EP, et al (2009) Highly efficient neural conversion of human ES and iPS cells by dual inhibition of SMAD signaling. Nat Biotechnol 27: 275–280. 19252484 10.1038/nbt.1529 PMC2756723

[23] Ericson J, Muhr J, Placzek M, et al (1995) Sonic hedgehog induces the differentiation of ventral forebrain neurons: a common signal for ventral patterning within the neural tube. Cell 81: 747–756. 7774016 10.1016/0092-8674(95)90536-7

[24] Chen JK, Taipale J, Cooper MK, et al (2002) Inhibition of Hedgehog signaling by direct binding of cyclopamine to Smoothened. Genes Dev 16: 2743–2748. 12414725 10.1101/gad.1025302 PMC187469

[25] Taipale J, Chen JK, Cooper MK, et al (2000) Effects of oncogenic mutations in Smoothened and Patched can be reversed by cyclopamine. Nature 406: 1005–1009. 10984056 10.1038/35023008

[26] Gaspard N, Bouschet T, Hourez R, et al (2008) An intrinsic mechanism of corticogenesis from embryonic stem cells. Nature 455: 351–357. 18716623 10.1038/nature07287

[27] Ciani L, Salinas PC (2005) WNTs in the vertebrate nervous system: from patterning to neuronal connectivity. Nat Rev Neurosci 6: 351–362. 15832199 10.1038/nrn1665

[28] Otero JJ, Fu W, Kan L, et al (2004) Beta-catenin signaling is required for neural differentiation of embryonic stem cells. Development 131: 3545–3557. 15262888 10.1242/dev.01218

[29] Hirabayashi Y, Itoh Y, Tabata H, et al (2004) The Wnt/beta-catenin pathway directs neuronal differentiation of cortical neural precursor cells. Development 131: 2791–2801. 15142975 10.1242/dev.01165

[30] Aubert J, Dunstan H, Chambers I, et al (2002) Functional gene screening in embryonic stem cells implicates Wnt antagonism in neural differentiation. Nat Biotechnol 20: 1240–1245. 12447396 10.1038/nbt763

[31] Verani R, Cappuccio I, Spinsanti P, et al (2007) Expression of the Wnt inhibitor Dickkopf-1 is required for the induction of neural markers in mouse embryonic stem cells differentiating in response to retinoic acid. J Neurochem 100: 242–250. 17064353 10.1111/j.1471-4159.2006.04207.x

[32] ten Berge D, Kurek D, Blauwkamp T, et al (2011) Embryonic stem cells require Wnt proteins to prevent differentiation to epiblast stem cells. Nat Cell Biol 13: 1070–1075. 21841791 10.1038/ncb2314 PMC4157727

[33] Li XJ, Zhang X, Johnson MA, et al (2009) Coordination of sonic hedgehog and Wnt signaling determines ventral and dorsal telencephalic neuron types from human embryonic stem cells. Development 136: 4055–4063. 19906872 10.1242/dev.036624 PMC2778748

[34] Sato N, Meijer L, Skaltsounis L, et al (2004) Maintenance of pluripotency in human and mouse embryonic stem cells through activation of Wnt signaling by a pharmacological GSK-3-specific inhibitor. Nat Med 10: 55–63. 14702635 10.1038/nm979

[35] Ruby KM, Zheng B (2009) Gene targeting in a HUES line of human embryonic stem cells via electroporation. Stem Cells 27: 1496–1506. 19544466 10.1002/stem.73

[36] Schmittgen TD, Livak KJ (2008) Analyzing real-time PCR data by the comparative C(T) method. Nat Protoc 3: 1101–1108. 18546601 10.1038/nprot.2008.73

[37] Chiao E, Kmet M, Behr B, et al (2008) Derivation of human embryonic stem cells in standard and chemically defined conditions. Methods Cell Biol 86: 1–14. 18442641 10.1016/S0091-679X(08)00001-0

[38] Pruszak J, Sonntag KC, Aung MH, et al (2007) Markers and methods for cell sorting of human embryonic stem cell-derived neural cell populations. Stem Cells 25: 2257–2268. 17588935 10.1634/stemcells.2006-0744 PMC2238728

[39] Schaeren-Wiemers N, Gerfin-Moser A (1993) A single protocol to detect transcripts of various types and expression levels in neural tissue and cultured cells: in situ hybridization using digoxigenin-labelled cRNA probes. Histochemistry 100: 431–440. 7512949 10.1007/BF00267823

[40] Reubinoff BE, Itsykson P, Turetsky T, et al (2001) Neural progenitors from human embryonic stem cells. Nat Biotechnol 19: 1134–1140. 11731782 10.1038/nbt1201-1134

[41] Patani R, Compston A, Puddifoot CA, et al (2009) Activin/Nodal inhibition alone accelerates highly efficient neural conversion from human embryonic stem cells and imposes a caudal positional identity. PLoS One 4: e7327. 19806200 10.1371/journal.pone.0007327 PMC2752165

[42] Miyoshi G, Fishell G (2012) Dynamic FoxG1 expression coordinates the integration of multipolar pyramidal neuron precursors into the cortical plate. Neuron 74: 1045–1058. 22726835 10.1016/j.neuron.2012.04.025 PMC3653132

[43] Cecchi C (2002) Emx2: a gene responsible for cortical development, regionalization and area specification. Gene 291: 1–9. 12095673 10.1016/s0378-1119(02)00623-6

[44] Sessa A, Mao CA, Hadjantonakis AK, et al (2008) Tbr2 directs conversion of radial glia into basal precursors and guides neuronal amplification by indirect neurogenesis in the developing neocortex. Neuron 60: 56–69. 18940588 10.1016/j.neuron.2008.09.028 PMC2887762

[45] Gotz M, Stoykova A, Gruss P (1998) Pax6 controls radial glia differentiation in the cerebral cortex. Neuron 21: 1031–1044. 9856459 10.1016/s0896-6273(00)80621-2

[46] Plachez C, Lindwall C, Sunn N, et al (2008) Nuclear factor I gene expression in the developing forebrain. J Comp Neurol 508: 385–401. 18335562 10.1002/cne.21645

[47] Casarosa S, Fode C, Guillemot F (1999) Mash1 regulates neurogenesis in the ventral telencephalon. Development 126: 525–534. 9876181 10.1242/dev.126.3.525

[48] Chambers I, Colby D, Robertson M, et al (2003) Functional expression cloning of Nanog, a pluripotency sustaining factor in embryonic stem cells. Cell 113: 643–655. 12787505 10.1016/s0092-8674(03)00392-1

[49] Niwa H, Miyazaki J, Smith AG (2000) Quantitative expression of Oct-3/4 defines differentiation, dedifferentiation or self-renewal of ES cells. Nat Genet 24: 372–376. 10742100 10.1038/74199

[50] Shu T, Butz KG, Plachez C, et al (2003) Abnormal development of forebrain midline glia and commissural projections in Nfia knock-out mice. J Neurosci 23: 203–212. 12514217 10.1523/JNEUROSCI.23-01-00203.2003 PMC6742120

[51] Panganiban G, Rubenstein JL (2002) Developmental functions of the Distal-less/Dlx homeobox genes. Development 129: 4371–4386. 12223397 10.1242/dev.129.19.4371

[52] Hevner RF, Shi L, Justice N, et al (2001) Tbr1 regulates differentiation of the preplate and layer 6. Neuron 29: 353–366. 11239428 10.1016/s0896-6273(01)00211-2

[53] Lai T, Jabaudon D, Molyneaux BJ, et al (2008) SOX5 controls the sequential generation of distinct corticofugal neuron subtypes. Neuron 57: 232–247. 18215621 10.1016/j.neuron.2007.12.023

[54] Itsykson P, Ilouz N, Turetsky T, et al (2005) Derivation of neural precursors from human embryonic stem cells in the presence of noggin. Mol Cell Neurosci 30: 24–36. 16081300 10.1016/j.mcn.2005.05.004

[55] Dimos JT, Rodolfa KT, Niakan KK, et al (2008) Induced pluripotent stem cells generated from patients with ALS can be differentiated into motor neurons. Science 321: 1218–1221. 18669821 10.1126/science.1158799

[56] Li XJ, Du ZW, Zarnowska ED, et al (2005) Specification of motoneurons from human embryonic stem cells. Nat Biotechnol 23: 215–221. 15685164 10.1038/nbt1063

[57] Li XJ, Hu BY, Jones SA, et al (2008) Directed differentiation of ventral spinal progenitors and motor neurons from human embryonic stem cells by small molecules. Stem Cells 26: 886–893. 18238853 10.1634/stemcells.2007-0620 PMC2707816

[58] Singh Roy N, Nakano T, Xuing L, et al (2005) Enhancer-specified GFP-based FACS purification of human spinal motor neurons from embryonic stem cells. Exp Neurol 196: 224–234. 16198339 10.1016/j.expneurol.2005.06.021

[59] Shi Y, Kirwan P, Smith J, et al.. (2012) Human cerebral cortex development from pluripotent stem cells to functional excitatory synapses. Nat Neurosci 15: 477–486, S471. 10.1038/nn.3041 PMC3882590 22306606

[60] Mukhopadhyay M, Shtrom S, Rodriguez-Esteban C, et al (2001) Dickkopf1 is required for embryonic head induction and limb morphogenesis in the mouse. Dev Cell 1: 423–434. 11702953 10.1016/s1534-5807(01)00041-7

[61] Houart C, Caneparo L, Heisenberg C, et al (2002) Establishment of the telencephalon during gastrulation by local antagonism of Wnt signaling. Neuron 35: 255–265. 12160744 10.1016/s0896-6273(02)00751-1

[62] Glinka A, Wu W, Delius H, et al (1998) Dickkopf-1 is a member of a new family of secreted proteins and functions in head induction. Nature 391: 357–362. 9450748 10.1038/34848

[63] Niehrs C (2004) Regionally specific induction by the Spemann-Mangold organizer. Nat Rev Genet 5: 425–434. 15153995 10.1038/nrg1347

[64] Shimizu T, Hibi M (2009) Formation and patterning of the forebrain and olfactory system by zinc-finger genes Fezf1 and Fezf2. Dev Growth Differ 51: 221–231. 19222525 10.1111/j.1440-169X.2009.01088.x

[65] Hashimoto H, Yabe T, Hirata T, et al (2000) Expression of the zinc finger gene fez-like in zebrafish forebrain. Mech Dev 97: 191–195. 11025224 10.1016/s0925-4773(00)00418-4

[66] Jeong JY, Einhorn Z, Mathur P, et al (2007) Patterning the zebrafish diencephalon by the conserved zinc-finger protein Fezl. Development 134: 127–136. 17164418 10.1242/dev.02705

[67] Glinka A, Wu W, Onichtchouk D, et al (1997) Head induction by simultaneous repression of Bmp and Wnt signalling in Xenopus. Nature 389: 517–519. 9333244 10.1038/39092

[68] Niehrs C (1999) Head in the WNT: the molecular nature of Spemann's head organizer. Trends Genet 15: 314–319. 10431193 10.1016/s0168-9525(99)01767-9

