# Supplementary figures and images for: Directed Differentiation of Human Embryonic Stem Cells into Corticofugal Neurons Uncovers Heterogeneous Fezf2-Expressing Subpopulations
Source: PLoS One. 2013 Jun 24;8(6):e67292. doi: 10.1371/journal.pone.0067292 (PMC3691138; doi:10.1371/journal.pone.0067292)

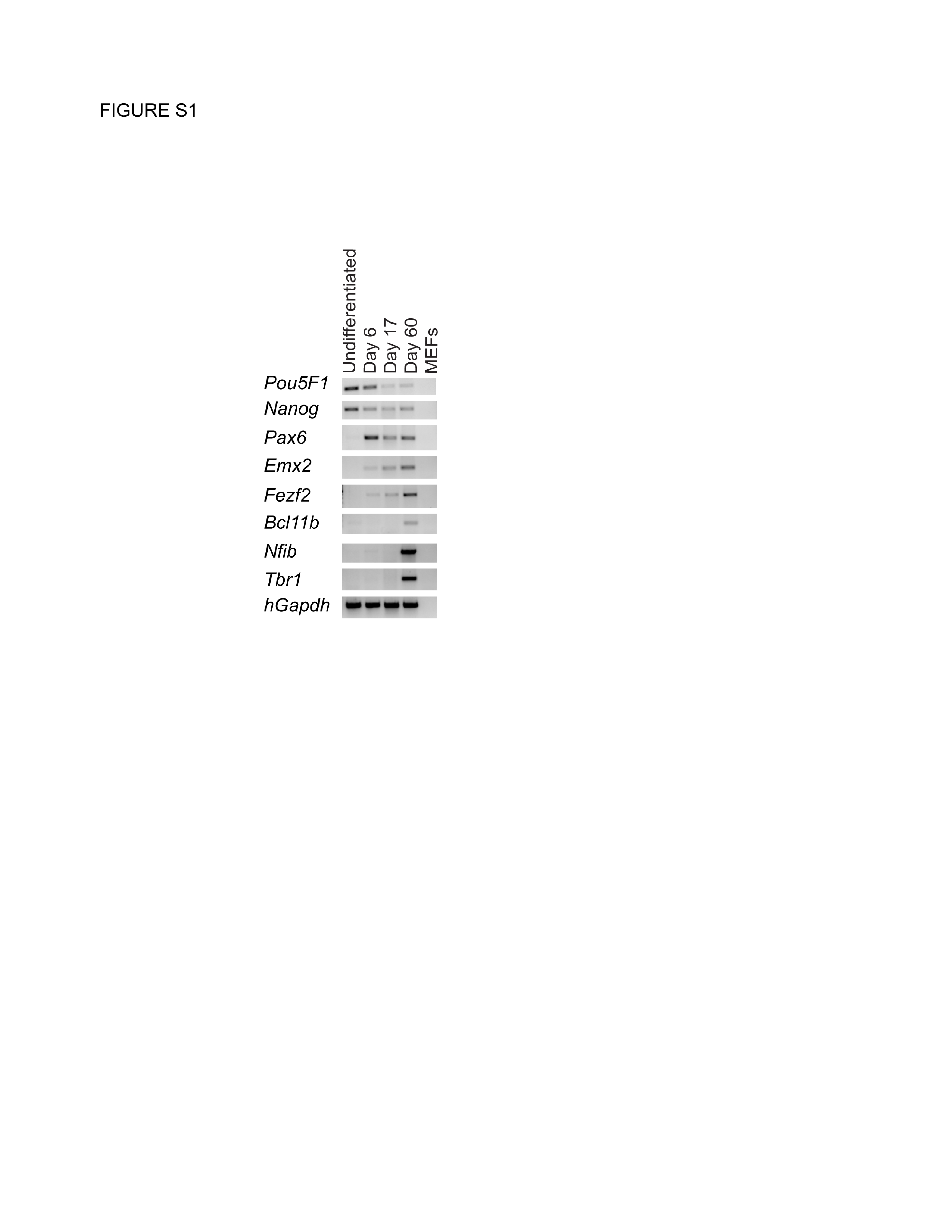

Supplement: Figure S1 — Reverse Transcriptase PCR (RT-PCR) shows expression of cortical cell markers in differentiated HUES 5 cells. Pluripotent markers Pou5F1 and Nanog are expressed in undifferentiated cells, their expression persists throughout differentiation albeit at lower levels. Radial glia marker, Pax6 is strongly expressed at day 6 and can still be detected at day 60. The neural progenitor marker Emx2 is detected starting at day 6 and its expression increases throughout differentiation until day 60. The corticospinal motor neuron Fezf2 is first detected at day 6 and is strongly expressed at day 60. Corticofugal neuron markers Bcl11b, Nfib and Tbr1 are all robustly expressed at day 60. None of the markers show expression in mouse embryonic fibroblasts (MEFs) control. (TIF) [file pone.0067292.s001.tif]

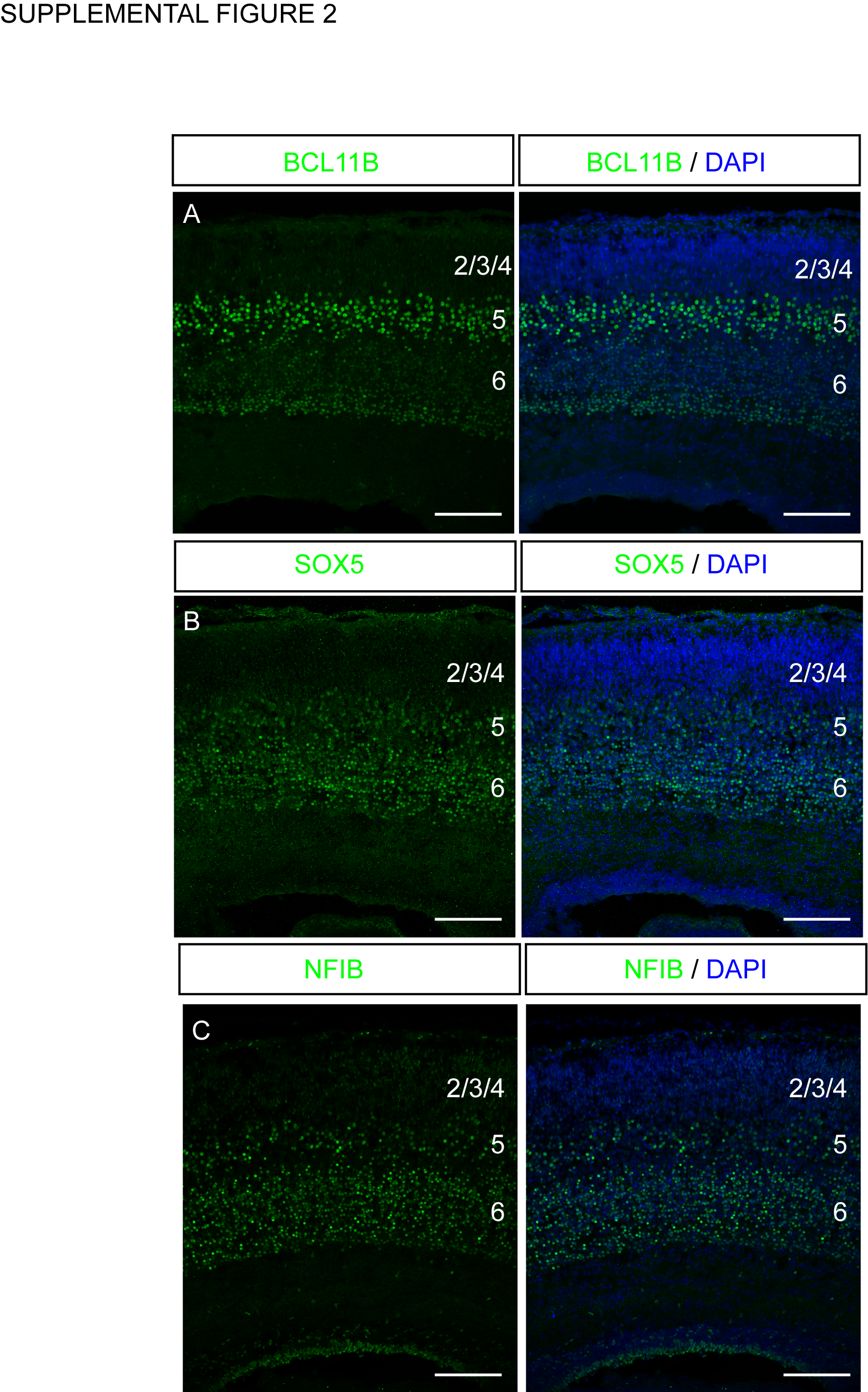

Supplement: Figure S2 — Immunohistochemistry of wild-type mouse cortex showing corticofugal neuron marker expression at P0. (A) Subcortical neuron marker BCL11B (green) is expressed strongly in layer 5 and weakly in layer 6 of the mouse cortex. DAPI indicates nuclear staining (blue) in all sections. (B) Corticofugal neuron marker SOX5 (green) is expressed in layer 5–6 of the mouse cortex. (C) Subcortical neuron marker NFIB (green) is expressed in layer 5–6 of the mouse cortex. Abbreviations: DAPI, 4′,6-diamidino-2-phenylindole; Scale bars: 150 µm. (TIF) [file pone.0067292.s002.tif]

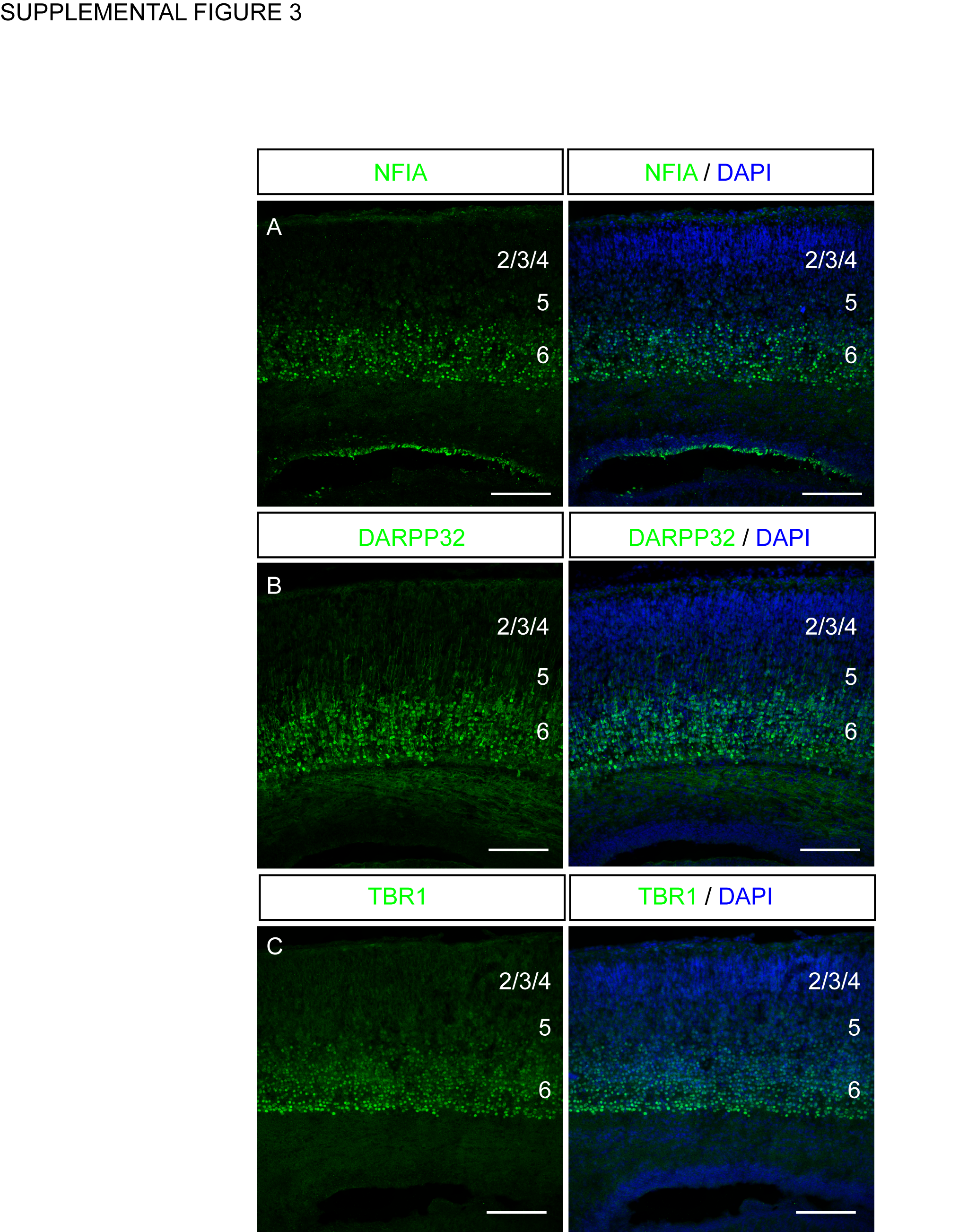

Supplement: Figure S3 — Immunohistochemistry of wild-type mouse cortex showing expression of layer 6 markers at P0. (A) NFIA (green) is expressed strongly in layer 6 of the mouse cortex. DAPI indicates nuclear staining (blue) in all sections. (B) DARPP32 (green) is strongly expressed in layer 6 of the mouse cortex. (C) Corticothalamic marker TBR1 (green) is expressed strongly in layer 6. Abbreviations: DAPI, 4′,6-diamidino-2-phenylindole; Scale bars: 150 µm. (TIF) [file pone.0067292.s003.tif]
