# Supplementary material for: Directed Differentiation of Human Embryonic Stem Cells into Corticofugal Neurons Uncovers Heterogeneous Fezf2-Expressing Subpopulations
Source: PLoS One. 2013 Jun 24;8(6):e67292. doi: 10.1371/journal.pone.0067292 (PMC3691138; doi:10.1371/journal.pone.0067292)
Supplement: Table S1 — Human and mouse RT-PCR primers. (DOCX) [file pone.0067292.s004.docx]

**Table S1. Human and mouse RT-PCR primers**

| **GENES** | **PRIMERS FORWARD** | **PRIMERS REVERSE** |
| --- | --- | --- |
| h*Pou5F1* | TGCAGAAAGAACTCGAGCAA | AGCTTCCTCCACCCACTTCT |
| h*Nanog* | TTGTGGGCCTGAAGAAAACT | ATCTGCTGGAGGCTGAGGTA |
| h*Pax6* | CGGTTTCCTCCTTCACAT | ATCATAACTCCGCCCATT |
| h*Emx2* | GCTTCTAAGGCTGGAACACG | TTGCGAATCTGAGCCTTCTT |
| h*Otx2* | GCTGGAAGATCTTGATGCG | AACCTCCCATGAGGCTGTAA |
| h*Foxg1* | CGGGCCAAGCTGGCCTTCAA | GCCGACGTGGTGCCGTTGTA |
| h*Fezf2* | TGCCAGGAGGCTCCGCAGAT | GCCGCTGTGGGTCAGCTTGT |
| h*Nfib* | AGCGCGCTCGGACTGCAA | AGCACGGGTGCTTCTTGCCA |
| h*Tbr1* | GGGCTCACTGGATGCGCCAAG | TCCGTGCCGTCCTCGTTCACT |
| h*Ascl1* | CATCTCCCCCAACTACTCCA | GAAAGCCATGTCTCTCAGGC |
| h*Gapdh* | CCTGACCTGCCGTCTAGAAA | GGTGGTCCAGGGGTCTTACT |
| m*Gapdh* | CGTCCCGTAGACAAAATGGT | TTGATGGCAACAATCTCCAC |
