# Supplementary material for: Directed Differentiation of Human Embryonic Stem Cells into Corticofugal Neurons Uncovers Heterogeneous Fezf2-Expressing Subpopulations
Source: PLoS One. 2013 Jun 24;8(6):e67292. doi: 10.1371/journal.pone.0067292 (PMC3691138; doi:10.1371/journal.pone.0067292)
Supplement: Table S2 — (DOCX) [file pone.0067292.s005.docx]

**Table S2**

**A. Human qPCR primers**

| **GENES** | **PRIMERS FORWARD** | **PRIMERS REVERSE** |
| --- | --- | --- |
| h*Fezf2* | TCCGCAGATGGCAAGCCCAAAA | TGCACACGAACGGTCTGGCTC |
| h*ActinB* | GCACAGAGCCTCGCCTTT | CACGATGGAGGGGAAGACG |
| h*Bhlhb5* | ATAGCGCCTGCGATTGTTTG | TTGGGACAGCGCTTTCTACC |
| h*Nfib* | AGCTGCTGGAAGTCGAACAT | TGAAGGTGGAGGTGGAGTTC |
| hNfia | GCAATGAGGAGGTCTTTACCCA | TAAAATGGCTCCTCACCAGGAC |
| h*Tbr1* | GACTCAGTTCATCGCCGTCA | TCGTGTCATAATTATCCCGAAATCC |
| hDarpp32 | CCTGAAGGTCATCAGGCAGT | GAGCCTCCATCTCTCTCGGA |
| h*Sox5* | TTCCAGCATGCTTACTGACCCTGA | GGGAGCCCGTCACTCTCCTCT |
| h*Pax6* | TCCGTTGGAACTGATGGAGT | GTTGGTATCCGGGGACTTC |
| h*Nestin* | AGGGCCTACAGAGCCAGATCGC | TGGAGCCACCGCCAGGTGTTT |
| h*Dlx5* | TTTGCCATTCACCATTCTCA | CGCTAGCTCCTACCACCAGT |
| h*Bcl11b* | TGTTGTGCAAATGTAGCTGG | GACTCAGGGTGAGGGTCAGA |

**B.** Mouse qPCR primers

| **GENES** | **PRIMERS FORWARD** | **PRIMERS REVERSE** |
| --- | --- | --- |
| *mFezf2* | AACCCAAAAACTTCACCTGCG | TTGCACACAAACGGTCTAGC |
| m*Actinb* | TTCTTTGCAGCTCCTTCGTT | ATGGAGGGGAATACAGCCC |
