# Supplementary material for: Directed Differentiation of Human Embryonic Stem Cells into Corticofugal Neurons Uncovers Heterogeneous Fezf2-Expressing Subpopulations
Source: PLoS One. 2013 Jun 24;8(6):e67292. doi: 10.1371/journal.pone.0067292 (PMC3691138; doi:10.1371/journal.pone.0067292)
Supplement: Table S3 — Primary Antibodies used in this study. (DOCX) [file pone.0067292.s006.docx]

Supplementary Table 3

Primary Antibodies used in this study

| **MARKERS/**  **GENES** | **SPECIES** | **MANUFACTURER** | **DESCRIPTION** |
| --- | --- | --- | --- |
| NFIA | Rabbit | Active Motif | Layer 6 |
| Nestin | Mouse | Millipore | Neural progenitor |
| Tuj1 | Mouse | Covance | Immature neurons |
| HuNu | Mouse | Millipore | Identifying cells of human origin |
| GFP | Chicken | Life Technologies | Identifying transplanted h*Fezf2* cells |
| NFIB | Rabbit | Active Motif | Layer 5-6 |
| TBR2 | Rabbit | Abcam | Neural progenitor |
| PAX6 | Rabbit | Covance | Neural progenitor |
| CTIP2/BCL11B | Rat | Abcam | Layer 5-6 cortical neurons |
| TBR1 | Rabbit | Abcam | Layer 5-6 cortical  neurons |
